# Response of Salivary Microbiota to Caries Preventive Treatment in Aboriginal and Torres Strait Islander Children

**DOI:** 10.1080/20002297.2020.1830623

**Published:** 2020-10-11

**Authors:** Emily Skelly, Newell W. Johnson, Kostas Kapellas, Jeroen Kroon, Ratilal Lalloo, Laura Weyrich

**Affiliations:** a Australian Centre for Ancient DNA, University of Adelaide, Adelaide, Australia; b School of Dentistry and Oral Health, Griffith University, Gold Coast, Australia; c Menzies Health Institute Queensland, Griffith University, Gold Coast, Australia; d Faculty of Dentistry, Oral and Craniofacial Sciences, King’s College London, London, UK; e Australian Research Centre for Population Oral Health, School of Dentistry, University of Adelaide, Adelaide, Australia; f School of Dentistry, The University of Queensland, Herston, Australia; g Department of Anthropology, The Pennsylvania State University, University Park, PA, USA

**Keywords:** Saliva, microbiota, caries, indigenous Australian, preventative, microbial ecology, oral health, children

## Abstract

A once-annual caries preventive (Intervention) treatment was offered to Aboriginal and Torres Strait Islander schoolchildren—a population with disproportionately poorer oral health than non-Indigenous Australian children—in the Northern Peninsula Area (NPA) of Far North Queensland (FNQ), which significantly improved their oral health. Here, we examine the salivary microbiota of these children (mean age = 10 ± 2.96 years; n = 103), reconstructing the bacterial community composition with high-throughput sequencing of the V4 region of bacterial *16S rRNA* gene. Microbial communities of children who received the Intervention had lower taxonomic diversity than those who did not receive treatment (Shannon, p < 0.05). Moreover, the Intervention resulted in further decreased microbial diversity in children with active carious lesions existing at the time of saliva collection. Microbial species associated with caries were detected; *Lactobacillus salivarius, Lactobacillus reuteri, Lactobacillus gasseri, Prevotella multisaccharivorax, Parascardovia denticolens*, and *Mitsuokella* HMT 131 were significantly increased (p < 0.05) in children with severe caries, especially in children who did not receive the Intervention. These insights into microbial associations and community differences prompt future considerations to the mechanisms behind caries-preventive therapy induced change;  important for understanding  the long-term implications of like treatment to improve oral health disparities within Australia.

*Trial registration: ANZCTR, ACTRN12615000693527. Registered 3 July 2015*, *https://www.anzctr.org.au/Trial/Registration/TrialReview.aspx?id=368750&isReview=true*

## Introduction

Aboriginal and Torres Strait Islander people (hereinafter respectfully referred to as ‘Indigenous’) makeup 2.8% of Australia’s population but have on average, over twice the number of decayed or missing teeth than non-indigenous Australian children [1]. Similarly, Indigenous adolescents suffer twice the rate of untreated dental caries than non-Indigenous Australians [1]. This not only precedes long-term systemic health problems, but manifests as pain and discomfort, causing difficulties in chewing and potential malnutrition, generating sleep disturbance, behavioural problems, lack of concentration and cooperation – all factors that can hinder learning, quality of life and overall well-being in young children [2,3].

Despite the importance of good oral health, the current trajectory appears to be worsening in Australian indigenous peoples [4], especially in rural communities that lack access to regular dental care. Ease of access to dental services significantly impacts the caries rate, as rural or remote Australian Indigenous children have poorer oral health relative to their urban counterparts [5]. As such, a 2004 oral health survey in the Northern Peninsula Area (NPA) of Far North Queensland (FNQ; located over 1,000 km north of the nearest city, Cairns), found the caries rate of 6- and 12-year-old Indigenous children to be double that of the state average and more than four times greater than that of the average Australian child [6].

In order to combat remoteness, a novel dental caries-preventive intervention was designed by Lalloo et al. [7] to decrease and/or slow the incidence of caries, with a focus on children living in remote communities. Preliminary results in the NPA reported that this preventive treatment significantly improved oral health after two consecutive annual treatments, resulting in a 29% decrease in caries incidence relative to children who did not receive treatment [8]. Despite the reduction in dental caries, the mechanisms that underpin this observation are not entirely understood. However, examining the microorganisms present in saliva – the most accessible, non-invasive, and child-friendly sampling strategy – can provide insights into the microbiota that not only contribute to dental caries, but those that may also be impacted by the environmental changes induced by caries-preventive treatments [9]. Salivary microbiota have previously been shown to be indicative of the number and severity of carious lesions, as well as to reflect the response to therapeutic interventions [10,11].

This investigation is especially pertinent to Indigenous populations, wherein current evidence suggests that the oral microbiota is distinct across populations, geographic locations, and/or ethnic identities [12], inclusive of the salivary microbiota [13,14]. As such, the analysis of dental plaque from Canadian First Nation children showed unique microbial abundances of cariogenic organisms in severe early childhood caries, and conversely, caries-free children were abundant in microbes not previously associated with oral health [15]. Moreover, a study of the dental calculus microbiota from Aboriginal Australian adults showed a distinctive microbial community from that found in non-Indigenous Australians, despite their shared periodontal disease state [16]. Such research highlights the importance of exploring both microbial differences between ethnic groups and how these specific microbial signatures may drive disease susceptibility.

Here, we aim to provide a first description of the salivary microbiota of Aboriginal and Torres Strait Islander children, who participated in a two-year-long trial of a caries-preventive intervention program. Using bacterial 16S rRNA amplicon sequencing, we investigated the impact of this novel preventive treatment on the salivary microbial community through the microbial community differences between children participating in the caries-preventive regime (herein referred to as the ‘Intervention’ group) to children untreated (the ‘Untreated’ group). Furthermore, we looked to explore how the salivary microbiota was associated with dental caries, highlighting potential biomarkers of dental caries within this remote population.

## Methods and materials

### Oral health survey and preventative intervention

The aim of the study was to evaluate ways to reduce the high prevalence of tooth decay in remote, rural Indigenous communities, which lack the appropriate social and health capital to provide traditional caries-preventive methods [7]. Evidence suggests that topical caries-preventive methods require several re-applications within a single year for efficacy [17,18]; limiting their application in remote and resource-constrained communities. Thus, this novel caries-preventative intervention was designed to be a sustainable and cost-effective approach, delivered within a single annual visit [7].

At baseline, in September/October 2015, oral health surveys were conducted by a team of four calibrated examiners in a school classroom, using mobile, reclinable dental chairs, with both fixed- and head-LED lights [19]. This consisted of a detailed head, neck, and dental clinical examination, alongside a questionnaire on basic demography (age and gender), residential history (i.e. exposure to fluoridated drinking water), perceptions surrounding general and oral health (oral health behaviours, attitudes, and knowledge, dental visits, and dietary information). Medical histories and a Statutory ‘Well Child Health Check’, as required by Queensland Health, the State public health authority, were simultaneously carried out. Children were then referred to a dentist or dental therapist in a community clinic, employed as part of the research team: those with caries cavities received a detailed treatment planning examination, including radiographs if indicated, and any necessary restorative care was offered. When completed they received the three-step preventative intervention described below. Children not requiring restorations received the three topical interventions at a single visit. Epidemiological screening and saliva sampling were repeated in school classrooms in September/October of 2016 and 2017.

All children attending school in the NPA of FNQ (two primary schools and one secondary) were invited to participate. As enrolment at the school varied each year and the study was designed to be completely inclusive, the number of participating children varied each year. Participants consented to the overall study, and then all participants could additionally accept or refuse the topical preventative intervention, as described in Kroon et al. [20] . The intervention implements a combination of three common topical treatments applied sequentially [7]. Firstly, teeth were swabbed with the antiseptic povidone-iodine (PVP-iodine, 10%, representing 0.5–1.0% available iodine). PVP-iodine has been shown to interfere directly with the binding ability of *mutans* streptococci to the tooth surface, as well as having broad antimicrobial activity [21,22]. Secondly, where indicated, fissure sealants were applied, predominantly to first permanent molars [23]. Finally, fluoride varnish was applied to all tooth surfaces, strengthening the enamel structure and promoting remineralisation, which is especially important in remote communities that lack water fluoridation [17,19,24]. Children who opted out of the Intervention due to cultural or logistical reasons acted as a natural comparison group (herein referred to as the ‘Untreated’). Of the 177 children who provided saliva in the final study year (2017), only children who attended all three surveys of the study (2015, 2016, 2017) were included in this analysis (n = 104; Intervention = 69, Untreated = 35). As saliva is sampled before the disinfectant and topical fluoride applications, children will have received two annual Intervention applications as of the 2017 sampling. Dental caries experience was recorded using the International Caries Detection and Assessment System (ICDAS; [25]). As ICDAS-II codes 1 and 2 represent the initial stage of demineralization (non-cavitated visual enamel changes), ICDAS‐II codes 0–2 were combined representing ‘caries-free’ and ICDAS-II codes 3–6 were defined as ‘caries-active’ [26].

### Sample collection

Stimulated saliva samples were collected before receiving the intervention, by chewing on paraffin wax for 5 min and dribbling into a sterile cup. Two mL of saliva was transferred into an OMNIgene•Oral OM-501 collection tube (DNA Genotek), which was stored at room temperature according to the manufacturer’s instructions. To control for potential airborne microbial contamination, samples of the air (n = 11) were collected by opening a blank collection tube in the school classroom, where the dental examination took place, for at least 1 min, both at the start and end of a day of saliva collection.

### DNA extraction, amplification, and sequencing

Sample extractions were performed in a dedicated clean facility designed for microbiome research at the University of Adelaide. Standard personal laboratory equipment included a laboratory coat, surgical facemask, shoe covers, and two layers of gloves (to allow frequent glove changes without skin exposure). All surfaces were cleaned prior to laboratory work with Decon 90 (Decon Laboratories Limited) and KlerAlcohol 70% v/v isopropyl alcohol (EcoLab Life Sciences). All extractions were prepared and completed in still-air cabinets, which were cleaned with a 2% bleach (NaClO) solution and UV-treated for 30 min prior to beginning any work.

Prior to extraction, 200 µL of saliva was incubated at 50°C for 1 h. The total genomic bacterial DNA was extracted using the Roche High Pure PCR Template Preparation Kit (Roche Life Sciences), according to the manufacturer’s instructions. Two extraction blank controls (EBCs) were included for each extraction batch (2 EBCs per 22 saliva samples). Following extraction, all samples were amplified in triplicate alongside an additional PCR no-template control, using barcoded primers specific to the V4 region of the *16S rRNA* gene, primer 515 F (5ʹ-GTGCCAGCMGCCGCGGTAA – 3ʹ) and 806 R (5ʹ-GGACTACHVHHHTWTCTAAT-3ʹ), as described in Caporaso et al. (2011) . Each PCR reaction contained: 18.05 μL sterile H_2_0, 1 μL of DNA extract, 0.25 μL of Hi-Fi taq (Life Technologies), 2.5 μL of 10 x Hi-Fi reaction buffer (Life Technologies), 1 μL MgSO_4_ (50 mM), 0.2 μL dNTPs (100 mM), and 1 μL each of the forward and reverse primers (10 uM). Samples were amplified under the following conditions: 95°C for 6 min; 38 cycles of 95°C for 30 s, 50°C for 30 s, and 72°C for 90 s; and a final step, 60°C for 10 min.

PCR triplicate products from each individual were pooled to a final volume of 75 μL and visualised by electrophoresis on a 2.5% agarose gel to check for size and quality of a representative sample. Samples were prepared for high-throughput sequencing by quantification on a fluorometer using a High Sensitivity dsDNA reagent kit (Qubit 2.0, Life Technologies), and pooled at equimolar concentrations for a normalized 5 nmol/L, before purification using AMPure cleanup (Ampure, Agencourt Bioscience). DNA sequencing and final quantification were completed at the Australian Genome Research Facility Ltd., Adelaide, across three 150 bp paired end MiSeq runs (Illumina).

### Data processing and statistical analyses

Raw Illumina BCL files were processed through BCL2fastq (v. 1.8.4; Illumina) to produce three fastq files (forward, barcodes, and reverse sequences). Metagenomic data were imported into the open-source QIIME2 platform (v. 2018.8 [27]) using the Earth Microbiome Project protocol for paired-end reads. Raw multiplexed paired-end fastq files were demultiplexed using barcodes through the q2-demux plugin, then denoised and quality-filtered using the Deblur plugin [28] (via q2-deblur). Sequences were truncated to 120 bp based on the median quality score. One saliva sample was removed from downstream analysis due to extremely low sequencing depth of 68 sequences (Sample ID: Bam17.200), leaving 103 samples for downstream analysis. MiSeq runs were merged (via q2-merge) and with the remaining samples, sequences were aligned using mafft plugin [29] to create a masked sequence alignment, removing highly variable positions (via q2-alignment). Phylogeny was constructed using fasttree [30] (via q2-phylogeny). Sequences assigned to chloroplast and mitochondria were removed with taxonomy-based filtering (via q2-taxa), assigning sequences to the Greengenes database (v. 13.8 [31]), prior to statistical analysis. QIIME (v. 1.9.1 [27]) was used for statistical analysis, exporting the merged feature table as a BIOM v2.1.0 formatted file and phylogeny artefact as a newick formatted file (via q2-export).

After importing files into QIIME, all statistical analyses were calculated at rarefaction depth of 19,255 sequences (the lowest sequencing depth of any sample). Alpha diversity metrics were computed using Shannon, observed species, and Chao1 indices (main text reporting Shannon; via *alpha_diveristy.py*) with significant group differences determined by nonparametric t-test, with 999 permutations (via *compare_alpha_diveristy.py*). Beta diversity analyses were completed using Bray-Curtis dissimilarity, binary Jaccard, weighted and unweighted UniFrac (main text reporting congruent Bray-Curtis results; via *beta_diveristy_through_plots.py*). Anosim analysis of similarities, and adonis permutational multivariate analysis of variance were used to test significant differences across sample groups, with 999 permutations (via *compare_catergories.py*). To achieve best possible species-level identification, taxonomic group differences were determined using Kruskal–Wallis nonparametric ANOVA calculated at the feature level (via *group_significance.py*), then assigned to three different reference databases: Greengenes (v. 13.8 [32]), Human Oral Microbiome Database (HOMD; v. 15.1 [33]) and the ribosomal database SILVA (132 release [34]). All main text reported species are HOMD assignments based on the specificity and confidence statistics. Significant differences of alpha and beta-diversity metrics were assessed using Bonferroni corrected p-values <0.05, where Kruskal-Wallis taxa significance was assessed using FDR corrected p-values <0.05.

### Figure construction

The R package phyloseq [35] was used to import OTU table, taxonomy tsv file, and sample metadata exported from QIIME2, and imported into RStudio [36]. After merging into the phyloseq object, using the microbiome R package, the object was transformed to compositional data [37], and ordinated for principle coordinate analysis using Bray-Curtis dissimilarity.

## Results

### Oral microbiome signal recovered from saliva

*16S rRNA* gene amplicons from all samples (total, n = 146; saliva, n = 103; sample controls, n = 33), after data trimming and quality filtering, produced a total of 6,991,276 sequences, with saliva samples contributing to 96.76% of the total sequences [SI Table 11]. Each saliva sample (n = 103) produced an average of 65,678 sequences (SD = 51,278, range 19,255–551,410). All sequences clustered into 1,221 features (i.e. the QIIME2 identifier for sub-operational taxonomic units or amplicon sequence variants). Of these, 1,056 features were only found in the salivary samples, and 165 features were unique to blank control samples.

**Figure 1.**
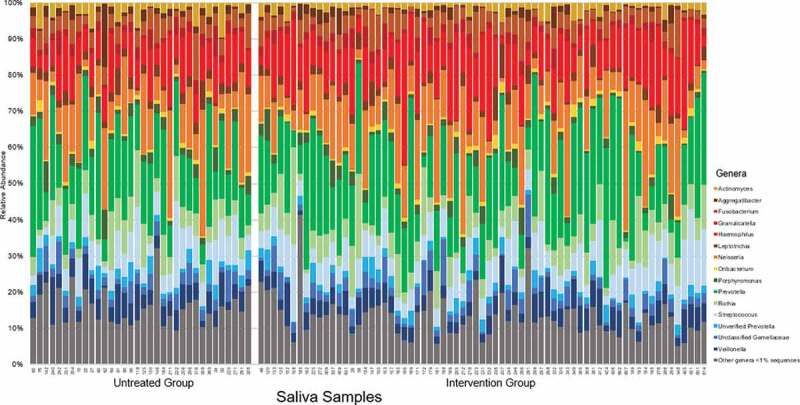
Relative abundance of the dominate genera (> 1% of total sequences) of saliva samples (n = 103) Each bar represents an individual saliva sample, showing similarities in the taxonomy of genera-level microbial composition between individual samples and treatment groups. Saliva samples had an average sequencing depth of 65,678 sequences after quality filtering (ranging from 19,255–551,410 sequences).

A total of 14 phyla, 23 classes, 42 orders, 76 families, and 119 genera were detected from 103 saliva samples. As expected, the most abundant phyla were *Proteobacteria* (average 29% of total sequences), *Bacteroidetes* (26%), *Firmicutes* (25%), *Actinobacteria* (11%), *Fusobacteria* (8%), and *Spirochaetes* (1%). From the 119 genera detected, 15 genera were dominant with a mean relative abundance >1% of the total sequences; these genera had a combined average contribution of 88.9% of the sequences identified in each sample: *Prevotella* (19.3%), *Neisseria* (13.1%), *Haemophilus* (12.5%), *Streptococcus* (9.2%), *Rothia* (7.4%), *Veillonella* (5.1%), *Fusobacterium* (4.6%), unclassified genera of family *Gemellaceae* (3.5%), *Actinomyces* (2.4%), *Granulicatella* (2.4%), *Porphyromonas* (2.4%), unverified *Prevotella* (2.3%), *Leptotrichia* (2.2%), *Aggregatibacter* (1.5%) and *Oribacterium* (1%) (Figure 1). There were no phyla or genera associated with age, dentition, or gender [see Supplementary Materials].

### Microbiota in blank controls is distinct from salivary samples

**Figure 2.**
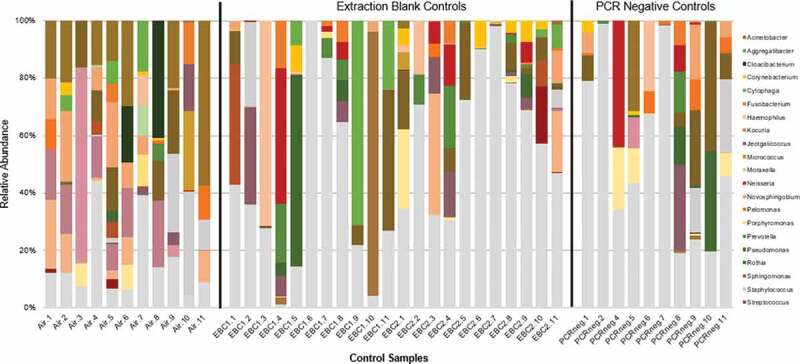
Relative abundance of the dominate genera (>1% of total sequences) of control samples (n = 43) Each bar represents a single sample; genera contributing more than 1% of total sequences are coloured, showing the variation of taxonomy and contamination content within the control samples. Controls have an average sequencing depth of 5,265 after quality filtering (ranging from 18⎯49,953 sequences).

Blank control samples (extraction blank controls (EBCs), PCR negatives, and air filter controls; total n = 43) contributed to a total of 226,401 sequences (3.24% of the total sequences), with an average of 5,265 sequences per sample (SD = 9821,62, range 18–49,953). Blank control samples shared only 280 overlapping features with salivary samples; likely due to reagent contamination and/or cross-contamination [38,39] . The control samples predominantly contained phyla *Proteobacteria* (mean relative abundance 46% of total sequences), *Firmicutes* (25%), *Actinobacteria* (15%), *Bacteroidetes* (6%), *Fusobacteria* (1%), *Cyanobacteria* (1%), and *Chloroflexi* (1%). There were 17 assigned genera with a mean relative abundance greater than (1%), and the top five dominating genera, *Staphylococcus* (mean relative abundance of 10.3% of the total sequences), *Acinetobacter* (7.7%), *Pseudomonas* (7%), *Novosphingobium* (6.2%), and *Micrococcus* (5.8%), are all known laboratory contaminants (Figure 2) [38] .

### Intervention treatment associated with decreased microbial diversity

**Figure 3.**
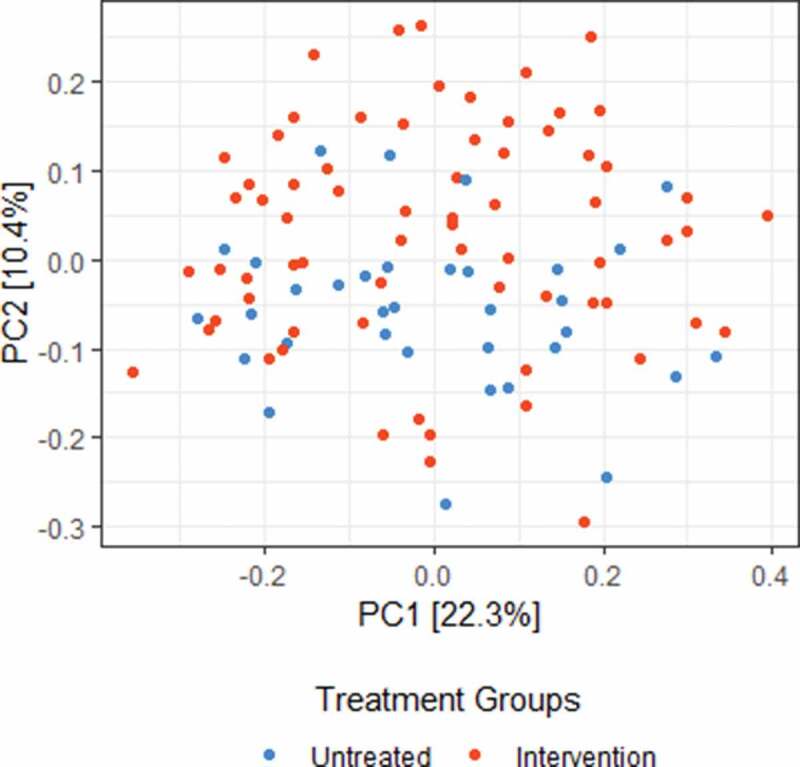
Intervention treatment had a limited impact upon the salivary microbial community structure Two-dimension (PC1 vs PC2) Principle coordinate analysis (PCoA) plot from Bray-Curtis beta-diversity distance matrices at the feature level. No significance was detected between the salivary microbial composition of untreated (n = 34) and intervention (n = 69) children, p > 0.05, suggesting a limited impact of the intervention upon the salivary microbial community structure.

To investigate the impact of the intervention on the salivary microbiota, we first analysed the differences in microbial species diversity between treatment groups (untreated (n = 34) vs intervention (n = 69)). We found individuals of the Untreated group had significantly higher microbial diversity compared to the Intervention group (Shannon, p = 0.001, t = 3.5), suggesting this treatment induced a reduction in the species diversity and richness. However, this difference was not accompanied by a change in microbial community structure (Bray-Curtis, adonis, p = 0.05, R2 = 0.018, anosim, p = 0.70, R = −0.019), suggesting that the Intervention minimally impacts the overall community structure between treatment groups (Figure 3). Further analysis of beta-diversity metrics can be found in the Supplementary Materials .

Kruskal–Wallis detected three species with significantly lower relative count in the Intervention group: *Lactobacillus salivarius* (Kruskal–Wallis, p = 0.04, t = 15.42), Unassigned *Selenomonas* (Kruskal–Wallis, p = 0.04, t = 14.85), and *Actinomyces* sp. HMT 896 (Kruskal–Wallis, p = 0.04, t = 14.78) [SI Table 2]. The lower count of caries-associated *L. salivarius* [40,41], alongside the lower microbial diversity of the Intervention group, suggests the Intervention may have had an impact on the microbial community associated with carious lesions present at the time of sampling.

### Presence of caries is not associated with changes in the oral microbiota

Given previous work identifying signals of dental caries in salivary microbial communities [42,43]; we first tested if the significance of carious lesions, as defined by the merged code ICDAS system [44], was linked with changes in the salivary microbial communities, regardless of treatment group. Microbial communities did not differ in species diversity between caries-free children (ICDAS scores of 0–2, i.e. showing no obvious sign of local enamel breakdown) and caries-active children (ICDAS scores = 3–6; Shannon, p = 0.40, t = 0.82).

Additionally, the presence or absence of caries did not have an effect on the microbial composition (Bray-Curtis, adonis; p = 0.028, R^2^ = 0.019); the variation between groups was also not significantly associated with the microbial community structure found between children with or without dental caries (Bray-Curtis, anosim, p = 0.13, R = 0.04). However, at the time of sampling, 85% of children in the Untreated group had carious lesions, relative to 55% of children in the Intervention group (Table 1). Therefore, we needed to account for the treatment group in the analysis of caries-associated microbial communities to determine the Intervention impact upon microbes associated with dental caries.

**Table 1.** Demographic information of sampled NPA children.

	Age (X̅ years ± SD)	Gender (Male/Female, n (%))	Caries (Active/Free, n (%))	Dentition (Mixed/All Permanent, n (%))
Intervention	10.43 ± 2.95	27/42 (39/61)	38/31 (55/45)	38/31 (55/45)
Untreated	9.6 ± 2.75	11/23 (32/68)	29/5 (85/15)	22/12 (65/35)
**Total**	10 ± 2.96	38/65 (37/63)	67/36 (67/35)	60/43 (58/42)

### Intervention differentially influences oral microbiota according to caries status

To explore how the Intervention impacted microbial communities associated with caries development, we first examined children according to their caries status (Table 1). In caries-free (CF) children, we found that the microbial communities did not differ in species diversity or richness between treatment groups (CF Intervention group (n = 31) vs CF Untreated group (n = 5); Shannon, p = 0.33, t = −1). Moreover, there were no significant differences in microbial community structure between treatment groups (Bray-Curtis, adonis, p = 0.54, R^2^ = 0.026; anosim, p = 0.9, R = −0.15). These results may suggest that the oral microbial communities in children with good oral health, *ab initio*, are not influenced by the Intervention – despite the use of broad-spectrum antimicrobials. However, due to the uneven sample groups and lack of radiographs to ascertain the caries-free status [45], these results should be interpreted with caution.

Next, we examined only children with active caries. Caries-active (CA) children who underwent the Intervention had significantly lower microbial diversity and richness relative to the CA Untreated group (CA Intervention (n = 38) vs. CA Untreated (n = 29); Shannon, p = 0.003, t = 3.42), which supports that the Intervention may have impacted the microbial ecology within active dental caries. However, we were unable to detect significant differences between the microbial community structure between CA Intervention or CA Untreated groups (Bray-Curtis, adonis, p = 0.06, R^2^ = 0.024; anosim, p = 0.377, R = 0.005).

### Severity of caries is related to oral microbial composition

As there was limited impact of presence or absence of dental caries on the microbial diversity, we looked to examine how caries severity may influence the oral microbiota – regardless of treatment – by combining all children into three levels of caries: None (i.e. no carious lesions detected, ICDAS-II score of 0–2 (n = 36)), Moderate (score 3–4; n = 33), and Severe (score 5–6; n = 34) (Table 2). In all children, regardless of treatment group, we were able to identify seven species significantly associated with increasing caries severity using Kruskal–Wallis [SI Table 9]: *L. salivarius* (p = 0.0007, t = 27.16), *Lactobacillus reuteri* (p = 0.0007, t = 27.16), *Lactobacillus gasseri* (p = 0.002, t = 24.53), *Prevotella multisaccharivorax* (p = 0.015, t = 19), *Streptococcus mutans* (p = 0.015, t = 18.94), *Parascardovia denticolens* (p = 0.015, t = 18.77), and *Mitsuokella* HMT 131 (p = 0.015, t = 18.46). Furthermore, four of these species, the three *Lactobacillus* species and *P. multisaccharivorax*, were not associated with caries until after the tooth enamel began to break down, supporting community changes with caries that could not be statistically detected with alpha diversity metrics. Interestingly, we were also able to detect a decrease of an unassigned *Treponema* species associated with increasing caries severity (Kruskal Wallis, p = 0.04, t = 15.9), potentially suggesting a positive relationship of this microbe to oral health.

**Table 2.** Active caries severity distribution of NPA children by treatment group, based on the merged International Caries Detection and Assessment System (ICDAS) score.

Active caries severity	Intervention group, n (%)	Untreated group, n (%)	Total, n (%)
None (ICDAS score 0–2)	31 (45%)	5 (15%)	36 (35%)
Moderate (ICDAS score 3–4)	22 (32%)	11 (15%)	33 (32%)
Severe (ICDAS score 5–6)	16 (23%)	18 (53%)	34 (33%)

### Intervention differentially impacts taxa associated with severe caries

**Figure 4.**
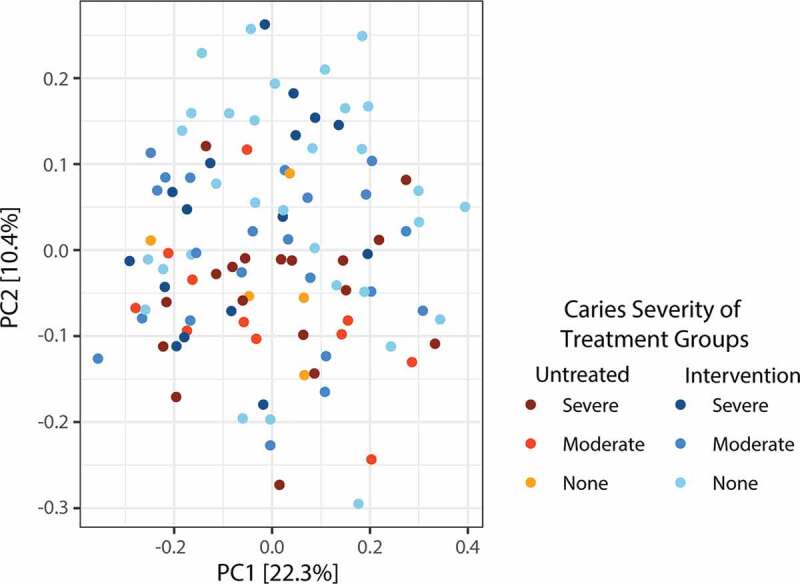
Limited impact of present caries or severity upon overall microbiota composition. Two-dimensional (PC1 vs PC2) principle coordinate analysis (PCoA) plot from Bray-Curtis beta-diversity distance matrices at the feature level. No significance was detected between the salivary microbial composition of different caries severities (Bray-Curtis adonis, p = 0.13, R^2^ = 0.026; anosim, p = 0.14, R = 0.014). The low-abundant taxa appear to be most indicative of caries severity and the influence of intervention treatment, suggesting saliva may have limited resolution as a caries detection strategy.

Lastly, we explored whether the Intervention treatment impacted the microorganisms linked to severity of active caries. We partitioned the samples into six groups based on caries severity of merged ICDAS scores and treatment groups (Table 2; Figure 4). Kruskal-Wallis detected that six of the seven taxa previously associated with active caries severity in all children (SI Table 9) were again significantly associated when accounting for treatment group (SI Table 10): three *Lactobacillus* species, *P. multisaccharivorax, P. denticolens*, and *Mitsuokella* HMT 131 (Table 3; p < 0.014, t > 25.95). Chiefly, *L. salivarius, L. reuteri*, and *L. gasseri* were detected at lower relative count at each level of caries within the Intervention group, compared to the Untreated group, suggesting that the Intervention was impacting upon *Lactobacillus* species present in carious lesions. In contrast, several species had an increased relative count with caries severity within the Intervention group, compared to untreated, including *P. multisaccharivorax, P. denticolens* and *Mitsuokella* HMT 131 (Table 3). Yet, some taxa, such as *Corynebacterium* and *Leptotrichia*, were only detected within the Untreated group, potentially indicative of the reduced microbial diversity seen with the Intervention. Overall, these results highlight the specificity of the Intervention to particular caries-associated bacterial taxa and allow us to identify a number of potential salivary biomarkers indicative of increasing caries severity within our remote NPA population.

**Table 3.** Nine species detected significantly different (Kruskal–Wallis test) between treatment groups when accounting for dental caries severity.

**HOMD (v. 15.1) output**				**Untreated** **(Mean relative count)**			**Intervention** **(Mean relative count)**		
**Species Assignment**	**Assigned Confidence**	**Test-Statistic**	**FDR** **p-value**	**ICDAS 0–2**	**ICDAS 3–4**	**ICDAS 5–6**	**ICDAS 0–2**	**ICDAS 3–4**	**ICDAS 5–6**
*Lactobacillus salivarius*	1.00	42.35	**5.28E-05**	0	0	10.22	0	0	2.56
Unassigned *Corynebacterium*	1.00	39.58	**9.56E-05**	1.40	0	0	0	0	0
Unassigned *Lactobacillus*	1.00	35.49	**0.00034**	0	0	6.44	0	0	1.75
*Prevotella multisaccharivorax*	1.00	35.35	**0.00034**	0	0	20.94	0	0.18	69.63
*Prevotella multisaccharivorax*	1.00	31.17	**0.00183**	0	0.18	33.61	0.16	2.68	11.06
*Mitsuokella* HMT 131	1.00	29.22	**0.0037**	0	0	5.5	0.06	0.09	5.75
*Lactobacillus gasseri*	0.95	25.95	**0.0138**	0	0.36	12.44	0	0.23	5.94
*Leptotrichia* sp. HMT 225	0.81	25.59	**0.0142**	0	1.18	0	0	0	0
*Parascardovia denticolens*	0.97	23.04	**0.0388**	0	0	7.06	0.06	0.73	5.31
*Streptococcus mutans*	1.00	21.49	0.0606	7.35	0.69	33.00	32.36	71.50	10.00

Bolded p-values are FDR corrected and significant p < 0.05. Unique 16S rRNA sequences were assigned species taxonomy using the Human Oral Microbiome Database (HOMD; v. 15.1; Chen et al. 2010), after testing for significance. Additional sequence information and species assignments are reported in SI Table 10 .

## Discussion

To our knowledge, this is the first study to investigate the oral microbiota of Aboriginal and Torres Strait Islander children of Australia, as well as the first to investigate long-term whole-community changes to the salivary microbiota in response to a caries-preventative treatment, and thus limited literature currently exists to interpret whole-community changes in saliva. Our results show that children who received the Intervention experienced a loss in species diversity (i.e. richness), which is typically indicative of an ecological disturbance; a discrete event causing the loss of microorganisms and an alteration to community structure [46]. Antimicrobial treatments are usually linked with the depletion of one or several specific taxa, reducing microbial diversity in the short term [47]. However, antimicrobial insults on the salivary microbiota appear transient, lasting around one week, with a near-complete recovery of the microbial community after a month [11,48]. For children in the NPA of FNQ, the repeated annual application of the Intervention appears to be driving a more permanent change and/or an incomplete recovery of the initial microbial community state. However, as we investigated saliva after two annual applications, further work is required to investigate both the immediate and longitudinal impact of a single Intervention application.

While conventional ecological theory suggests reduced species diversity may limit resilience to ecological instability or invading pathogens [49], this may not be pertinent to oral health, where greater microbial diversity has been observed with oral disease, compared to that of healthy individuals [43,50]. This observation is supported by our results, as we observed lower microbial diversity in children who received the Intervention, and despite the presence of dental caries, was overall linked to improved oral health within this population [8]. On a shorter timescale, Belstrøm et al. showed a corresponding increase in salivary microbial diversity with the cessation of oral hygiene and accumulation of dental plaque, which was then reversed after the uptake of oral hygiene [51]. This might suggest that decreased microbial diversity induced by the Intervention is symptomatic of preventative mechanisms supporting oral health. The direct mechanisms behind the reduction in dental caries with the Intervention would be best elucidated by the study of the dental plaque microbial biofilm. However, as saliva can detach plaque layers and promote plaque growth, saliva acts as both a microbial reservoir and mode of microbial transmission [52].

Subsequently, we hypothesise the differences detected in salivary diversity from the Intervention may be indicative of similar modifications to the dental plaque microbial ecology towards a state supportive of oral health. How this occurs could not be concluded by our results and requires further study. It is possible the Intervention may be acting upon the microbial community functionality or indirectly impacting the environmental variables that define the microbial ecology [49]. Understanding the impact of these treatments on the overall microbial ecology of the mouth is critical to understanding the long-term implications, benefits, or risks, associated with novel dental treatments. Longitudinal tracking of the possible downstream effects from an initial ecological shift has often been disregarded in oral health research and needs to be included in studies moving forward, especially in Indigenous populations that may retain unique microorganisms [53].

In this study, the bacterial species associated with dental caries – detected at a significantly greater count in children with severe dental caries – represent a very small proportion of the overall salivary microbiota. As such, these species were likely to shed from the plaque microbiota of carious lesions [51] and disrupted by paraffin wax for salivary collection [54]. Thus, while oral microbes in a planktonic state are not usually regarded as direct casual agents of dental caries, understanding specific bacteria associated with the presence of active caries can facilitate prevention and treatment [9], especially against those associated acidogenic and acidophilic species that are more likely to contribute to the caries process. We found that *L. salivarus, L. reuteri, L. gasseri, S. mutans, P. multisaccharivorax, P. denticolens*, and *Mitsuokella* HMT 131 were all significantly increased within the salivary microbiota of  children with severe carious lesions. *L. salivarius* and *P. denticolens* have been previously detected in saliva of individuals with progressive carious lesions within multiple populations [40,41,55,56]. *Lactobacillus* species are hypothesized to supervene the formation of the carious lesion, supporting downstream enamel demineralisation by more acidogenic species, such as *S. mutans* [57]. The functional repertoire of *Lactobacillus* species (i.e. the ability to thrive in a low pH environment and produce lactic acids [58]) suggests that its presence supports the development and severity of carious lesions. It is possible that *Lactobacillus* species play a more significant role in caries development within this population than previously appreciated.

While *Mitsuokella* HMT 131 has not previously been associated with dental caries, it has been detected and associated with subgingival plaque of periodontitis and dental root canals [59,60]. Similarly, *P. multisaccharivorax* has been previously associated with a widerange of oral diseases, including severe early-childhood caries, root caries, and periodontal disease [61,62]. As *Mitsuokella* and *Prevotella* species were associated with increasing caries severity and failed to respond to the intervention treatment, further research is needed to understand how to limit or control their presence. Further longitudinal work should explore the relationships between these microorganisms and the downstream potential to develop dental disease, which is significantly increased in Aboriginal and Torres Strait Islander people, both in childhood and later in life [63,64].

Despite both the cost-effectiveness and large-batch processing ease of samples with 16S rRNA, there are limitations in using amplicon sequencing to achieve a detailed understanding of the salivary microbial community and its role in caries. First, there are known biases in the *16S* gene for understanding the microbial species presence. While we used the V4 region and protocols used in the Human Microbiome Project (HMP) [65] – shown to have the highest quality classification accuracy [66] – the discrepancies between different variable regions and protocols across numerous salivary microbiota studies limit our ability to truly cross-compare microbial differences between this population and other studies. Second, sequencing with 16S rRNA also restricts the ability to describe increased or decreased ‘abundance’ of a particular microbial species associated with oral health or disease, as detection can be influenced by the number of 16S rRNA operon copies present in the bacterial genome [67]. Thus, only relative abundance can be discussed, which may not reflect the true biological ecosystem, although we used both the normalization and nonparametric Kruskal-Wallis test to circumvent some of these issues [68]. Lastly, the choice of reference database will influence the taxonomic assignment (as seen in SI Tables 9 & 10). While the Greengenes database was popularised by the HMP, unfortunately, it has not been updated since May 2013 and is quickly becoming outdated. The HOMD database of oral microbes is also problematic; although it can more accurately classify microorganisms present in the oral environment (of predominantly urban-industrialised populations), this impedes assignments to any species not previously identified in the oral environment, and masks potentially novel species found in understudied populations of various cultural and environmental niches. SILVA has the opposite dilemma, wherein its assignment to various environmental niches is accurate, it has less specificity for oral taxa. We attempted to mitigate these ascertainment biases through the use of multiple databases for taxonomic identification. While shotgun metagenomic sequencing approaches will mitigate some of these effects, further exploration of the microbiota from underrepresented populations is still a key issue moving forward [69].

In summary, this novel caries-preventative intervention was associated with a loss of oral microbial diversity, alongside the improved oral health of children in the NPA of FNQ. Six unsuspected biomarkers for severe caries in this population of Aboriginal and Torres Strait Islander children were indicative of the selective impact of the Intervention upon caries-associated microorganisms, without significantly impacting the overall microbiota community. Lastly, this study also demonstrates the use of non-invasive saliva collection to assess links between the oral microbiota, dental disease, and caries treatment, providing key information to assist the development of better caries interventions and to assess longitudinal outcomes of caries prevention programs, especially within underrepresented Indigenous populations. At the most basic level, further research is needed to explore the oral microbial communities and how their recent alterations contribute to the oral health of Indigenous Australians, especially in relationship to the different rates of oral disease observed in those of non-Indigenous descent. Such work is required in the efforts to diminish the persisting oral health gap.

## Supplementary Material

Supplemental Material

## Data Availability

The bacterial 16S rDNA sequences reported in this paper have been deposited to QIITA (Study ID: 12572). All additional material is included in this published article.
