# Supplementary material for: Response of Salivary Microbiota to Caries Preventive Treatment in Aboriginal and Torres Strait Islander Children
Source: J Oral Microbiol. 2020 Oct 11;12(1):1830623. doi: 10.1080/20002297.2020.1830623 (PMC7586720; doi:10.1080/20002297.2020.1830623)
Supplement: Supplemental Material [file ZJOM_A_1830623_SM7695.docx]

Supplementary Results

**Response of Salivary Microbiota to Caries Preventive Treatment in Australian Indigenous Children**

Table of Contents:

[Index of Figures and Tables: 2](#_Toc50992690)

[Results 3](#_Toc50992691)

[*1.1. Age, dentition, and gender did not drive significant variation in salivary microbiota* 3](#_Toc50992692)

[*1.2. Intervention treatment associated with decreased microbial diversity* 4](#_Toc50992693)

[*1.3. Presence of caries is not associated with oral microbiota* 6](#_Toc50992694)

[*1.4. Investigating the impact of uneven sample groups* 7](#_Toc50992695)

[1.4.1. Intervention and Untreated groups 7](#_Toc50992696)

[1.4.2. Caries-Active Intervention and Caries-Active Untreated groups 10](#_Toc50992697)

[*1.5 Severity of caries is related to oral microbial composition* 12](#_Toc50992698)

[References 16](#_Toc50992699)

# Index of Figures and Tables:

[**SI Table 1.** Alpha diversity statistics, Shannon, Observed species, and Chao1, tests of significance for demographic variables. All p-values are Bonferroni corrected; no significance was detected (p < 0.05). 3](#_Toc50992593)

[**SI Table 2*.*** Beta diversity statistics, Bray-Curtis, binary Jaccard, weighted and unweighted UniFrac distance metrics, tests of significance for demographic variables. All p-values are Bonferroni corrected; bolded values indicate significance p < 0.05. 4](#_Toc50992594)

[**SI Figure 1. Alpha diversity box plots of three metrics: Observed species, Chao1, and Shannon.** Alpha diversity was significantly higher in the Untreated groups with all three metrics: Observed species, p = 0.004, t = 2.96; Chao1, p = 0.009, t = 2.77; and Shannon, p = 0.001, t = 3.5. 5](#_Toc50992595)

[**SI Table 3. Testing differences between even-numbered Treatment groups.** Alpha diversity measures of significant differences between even sample groups (Intervention (n = 34) vs Untreated (n = 34)), tested three different times after the removal of 35 random Intervention samples. All alpha diversity metrics with significance are bolded, Bonferroni corrected p-values < 0.05. 8](#_Toc50992596)

[**SI Table 4. Beta-diversity testing even-numbered Treatment groups.** Beta diversity measures of significant difference between even sample groups (Intervention (n = 34) vs Untreated (n = 34)), tested three different times after the removal of 35 random Intervention samples. All beta diversity metrics with significance are bolded, Bonferroni corrected p-values < 0.05. 8](#_Toc50992597)

[**SI Figure 2. Principle coordinate analysis (PCoA) plots from Bray -Curtis beta diversity distance matrices at the feature level. (A)** Removal of 35 random samples in Test 2 of Intervention (n = 34) vs Untreated (n = 34) induced a significant difference between samples groups (anosim, p = 0.03, R = 0.044), whereas **(B)** uneven sample groups of Intervention (n = 69) vs Untreated (n = 34) was found not significant (anosim, p = 0.70, R = -0.019). All samples are plotted in three-dimensional space relative to their dissimilarity to one another. 10](#_Toc50992598)

[**SI Table 5. Testing even-numbered sample groups.** Alpha diversity measures of significant differences between even sample groups (CA Intervention (n = 29) vs CA Untreated (n = 29)), tested three different times after the removal of nine random CA Intervention samples. All alpha diversity metrics with significance are bolded, Bonferroni corrected p-values < 0.05. 11](#_Toc50992599)

[**SI Table 6. Testing even-numbered sample groups.** Beta diversity measures of significant difference between even sample groups (CA Intervention (n = 29) vs CA Untreated (n = 29)), tested three different times after the removal of nine random CA Intervention samples. All beta diversity metrics with significance are bolded, Bonferroni corrected p-values < 0.05. 12](#_Toc50992600)

[**SI Table 7.** Alpha diversity statistics for caries severity groups, not accounting for impact of treatment group. Bonferroni corrected p-values < 0.05; no significance was detected. 13](#_Toc50992601)

# Results

## *1.1. Age, dentition, and gender did not drive significant variation in salivary microbiota*

Previous salivary research has identified microbial differences in saliva associated with age and dentition [1]. Therefore, we tested the impact of dentition (mixed-permanent dominant (n = 31), vs mixed-deciduous dominant (n = 29), vs all-permanent dentition (n = 43)), age group (ages 6–8 (n = 33), vs ages 9–13 (n = 54), vs ages 14–17 (n = 16)), as well as gender (male (n = 38) vs female (n = 65)) on microbial community composition and structure, and measured by alpha and beta diversity metrics, respectively. There was no support for compositional differences (alpha diversity) driven by any demographic (SI Table 1; Shannon, observed species, Chao1, p > 0.05, t (range) = -1.53–1.82). Moreover, there was no support for age or gender contributing to microbial community structure (beta diversity) as confirmed by Bray-Curtis, binary Jaccard metrics, and weighted UniFrac (SI Table 2).

| **Groups** | **Shannon** | | **Observed species** | | **Chao1** | |
| --- | --- | --- | --- | --- | --- | --- |
|  | T statistic | p-value | T statistic | p-value | T statistic | p-value |
| **Age groups** |  |  |  |  |  |  |
| ages 6–8 *vs* ages 9–13 | -0.867 | 1 | -0.617 | 1 | -0.739 | 1 |
| ages 6–8 *vs* ages 14–17 | 0.890 | 1 | 0.753 | 1 | 0.396 | 1 |
| ages 9–13 *vs* ages 14–17 | 1.824 | 0.246 | 1.355 | 0.519 | 1.085 | 0.813 |
| **Dentition groups** |  |  |  |  |  |  |
| all-permanent *vs* mixed-permanent dominant | -0.236 | 1 | -0.621 | 1 | -0.430 | 1 |
| mixed-deciduous dominant *vs* mixed-permanent dominant | -0.832 | 1 | -1.526 | 0.417 | -1.488 | 0.474 |
| mixed-permanent dominant *vs* all-permanent | -0.582 | 1 | -1.129 | 0.738 | -1.313 | 0.6 |
| **Gender** |  |  |  |  |  |  |
| male *vs* female | 0.721 | 0.479 | 0.873 | 0.369 | 0.833 | 0.397 |

**SI Table 1.** Alpha diversity statistics, Shannon, Observed species, and Chao1, tests of significance for demographic variables. All p-values are Bonferroni corrected; no significance was detected (p < 0.05).

However, differences between dentition groups as measured by unweighted UniFrac were significant (anosim; p = 0.04, R = 0.0638, adonis; p = 0.005, R^2^ = 0.0754; SI Table 2). Significant phylogenetic shifts with changing child dentition have been previously reported with support from weighted UniFrac [2], thus we could interpret the disparate weighted and unweighed UniFrac results to suggest changing dentition alters the abundances present in the microbial community. Therefore, caution should be used in the interpretation of unweighted UniFrac results, as they will be confounded by differences in dentition.

| **Groups** | **Bray-Curtis** | | | | **Binary Jaccard** | | | | |
| --- | --- | --- | --- | --- | --- | --- | --- | --- | --- |
|  | **Adonis** | | **Anosim** | | **Adonis** | | | **Anosim** | |
|  | R^2^ | p-value | R statistic | p-value | R^2^ | p-value | | R value | p-value |
| **Age groups** | 0.0151 | 0.80 | 0.0213 | 0.23 | 0.0217 | | 0.17 | 0.0512 | 0.06 |
| **Dentition groups** | 0.0115 | 0.98 | -0.0301 | 0.92 | 0.0247 | | 0.05 | 0.0220 | 0.16 |
| **Gender** | 0.0104 | **0.03** | -8.51E^-5^ | 0.49 | 0.0095 | | 0.05 | 0.0059 | 0.43 |
| **Groups** | **Weighted UniFrac** | | | | **Unweighted Unifrac** | | | | |
|  | **Adonis** | | **Anosim** | | **Adonis** | | | **Anosim** | |
|  | R^2^ | p-value | R statistic | p-value | R^2^ | p-value | | R value | p-value |
| **Age groups** | 0.0377 | 0.71 | -0.0243 | 0.78 | 0.0547 | | 0.22 | 0.0065 | 0.42 |
| **Dentition groups** | 0.0360 | 0.78 | -0.0230 | 0.76 | 0.0754 | | **0.005** | 0.0638 | **0.04** |
| **Gender** | 0.0219 | 0.66 | -0.0020 | 0.50 | 0.0431 | | 0.05 | 0.0366 | 0.13 |

**SI Table 2*.*** Beta diversity statistics, Bray-Curtis, binary Jaccard, weighted and unweighted UniFrac distance metrics, tests of significance for demographic variables. All p-values are Bonferroni corrected; bolded values indicate significance p < 0.05.

## *1.2. Intervention treatment associated with decreased microbial diversity*

Those in the Untreated group contained significantly higher microbial diversity compared to the Intervention group with all alpha diversity metrics (SI Figure 1; Shannon, p = 0.001, t = 3.5; Chao1, p = 0.009, t = 2.77; Observed species, p = 0.004, t = 2.96), suggesting a reduction in the microbial diversity and richness of saliva in some children who received treatment.


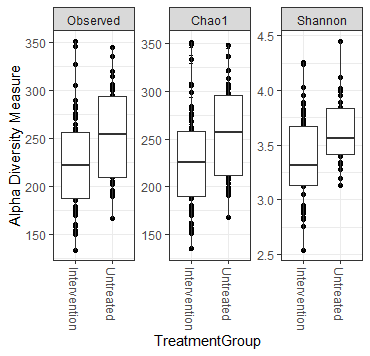


**SI Figure 1. Alpha diversity box plots of three metrics: Observed species, Chao1, and Shannon.** Alpha diversity was significantly higher in the Untreated groups with all three metrics: Observed species, p = 0.004, t = 2.96; Chao1, p = 0.009, t = 2.77; and Shannon, p = 0.001, t = 3.5.

However, this difference in diversity was not accompanied by a change in microbial community structure. While the adonis tests of three of four beta diversity measures suggest microbial community variation could potentially be explained by treatment (Untreated (n = 34) vs Intervention (n = 69); Bray-Curtis adonis, p = 0.04, R^2^ = 0.018; binary Jaccard adonis, p = 0.007, R^2^ = 0.016; weighted UniFrac adonis, p = 0.33, R^2^ = 0.011, unweighted UniFrac adonis, p = 0.02, R^2^ = 0.02), the microbial differences of the community structure between the treatment groups appear too subtle to statistically detect (Bray-Curtis anosim, p = 0.70, R = -0.019; binary Jaccard anosim, p = 0.70, R = -0.019, weighted UniFrac anosim, p = 0.78 , R = -0.023 , unweighted UniFrac anosim, p = 0.4, R = 0.011). Overall, these results suggest that while the Intervention appears to impact the microbial diversity, it has minimal impacts on the community structure of salivary microbiota between treatment groups.

## *1.3. Presence of caries is not associated with oral microbiota*

To test the influence of caries on the salivary microbial communities, we initially evaluated the differences in microbial diversity with the presence or absence of carious lesions, regardless of treatment group, using the merged code ICDAS system [3]. No significant differences in the microbial diversity were detected between caries-free children (CF (n = 35); ICDAS scores of 0–2, *i.e.* showing no obvious sign of local enamel breakdown) vs caries-active children (CA (n = 67); ICDAS scores = 3–6) calculated by alpha diversity metrics (Shannon, p = 0.40, t = 0.82; Observed species, p = 0.40, t = 0.73; Chao1, p = 0.46, t = 0.72).

Further examination revealed no significant differences in the microbial community structure between children with or without dental caries (Bray-Curtis anosim, p = 0.13, R = 0.04; binary Jaccard anosim, p = 0.10, R = 0.04; unweighted Unifrac anosim, p = 0.40, R = 0.0045; weighted Unifrac anosim, p = 0.70, R = -0.017), although significant variation in the microbial abundance associated with the presence or absence of caries could be detected using Bray-Curtis (adonis; p = 0.028, R^2^ = 0.019). However, binary Jaccard, unweighted and weighted UniFrac distance measures did not support any contribution of caries presence to microbial variation (binary Jaccard, adonis; p = 0.09, R^2^ = 0.0120; unweighted Unifrac adonis, p = 0.09, R^2^ = 0.015; weighted Unifrac adonis, p = 0.25, R^2^ = 0.0120). Overall, this suggests the presence or absence of carious lesions do not impact the salivary microbial community diversity or structure.

Next, to isolate the impact caries status may be contributing to treatment group differences, we investigated the microbial community within only CA children (CA Intervention (n = 38) vs. CA Untreated (n = 29)). Microbial diversity of CA Intervention children was significantly lower than the microbial diversity of CA Untreated group (Shannon; p = 0.006, t = 3.41, observed species; p = 0.024, t = 3.04, Chao1; p = 0.048, t = 2.70), showing that the microbes present within the salivary microbial community with active dental caries greater within children who did not receive treatment.

While the presence/absence measure of microbial diversity could explain some variation between treatment groups as tested with Binary Jaccard (adonis, p = 0.017, R^2^ = 0.02), this was not supported by unweighted UniFrac (adonis, p = 0.06, R^2^ = 0.0247), or by abundance based distance measures (Bray-Curtis adonis, p = 0.06, R^2^ = 0.024; weighted UniFrac adonis, p = 0.12, R^2^ = 0.0286). Moreover, the microbial community structure did not significantly differ between CA treatment groups, with no significant differences detected with any beta diversity distance measure (Binary Jaccard; anosim, p = 0.279, R = 0.02; Bray-Curtis anosim, p = 0.377, R = 0.005, unweighted UniFrac anosim, p = 0.25, R = 0.0142, weighted UniFrac anosim, p = 0.35, R = 0.0041). These results support an impact of Intervention treatment on community diversity that is not necessarily linked to caries status, yet the difference in species diversity is not driving significant changes to the overall microbial community.

##

## *1.4. Investigating the impact of uneven sample groups*

### 1.4.1. Intervention and Untreated groups

With the uneven sample groups between treatment groups and caries status, we wished to test whether the uneven sampling number was contributing to our results. Firstly, we tested the differences noted between children receiving the treatment (Intervention group; n = 69) and those who did not (Untreated group; n = 34). This was done by randomly assigning numbers to the larger treatment group (*i.e.* Intervention) and removing the first 35 samples (35 different individuals). Then alpha and beta diversity analysis was repeated to identity differences between even-numbered Intervention group (n = 34) and Untreated group (n= 34). This process was then repeated two more times, each time removing 35 different random samples to create a total of three different datasets (Test 1, Test 2, and Test 3).

All alpha diversity metrics, across the three different tests, were congruent with the findings seen in the unequal sample groups, supporting significantly greater microbial diversity in children who did not receive Intervention treatment (SI Table 3, p < 0.01).

|  | **Intervention (n = 34) vs Untreated (n = 34)** | | | | | |
| --- | --- | --- | --- | --- | --- | --- |
|  | **Shannon** | | **Observed species** | | **Chao1** | |
|  | T statistic | p-value | T statistic | p-value | T statistic | p-value |
| **Test 1** | 3.5806 | **0.002** | 2.6426 | **0.013** | 2.2962 | **0.027** |
| **Test 2** | 2.9665 | **0.004** | 2.7821 | **0.007** | 2.5464 | **0.018** |
| **Test 3** | 2.9616 | **0.011** | 2.7843 | **0.008** | 2.4522 | **0.015** |

**SI Table 3. Testing differences between even-numbered Treatment groups.** Alpha diversity measures of significant differences between even sample groups (Intervention (n = 34) vs Untreated (n = 34)), tested three different times after the removal of 35 random Intervention samples. All alpha diversity metrics with significance are bolded, Bonferroni corrected p-values < 0.05.

|  | **Intervention (n = 34) vs Untreated (n = 34)** | | | | | | | |
| --- | --- | --- | --- | --- | --- | --- | --- | --- |
|  | **Bray-Curtis** | | | | **Binary Jaccard** | | | |
|  | Adonis | | Anosim | | Adonis | | Anosim | |
|  | R^2^ | p-value | R statistic | p-value | R^2^ | p-value | R value | p-value |
| **Test 1** | 0.0276 | **0.033** | 0.0331 | 0.061 | 0.0184 | 0.084 | 0.0204 | 0.125 |
| **Test 2** | 0.0274 | **0.049** | 0.0443 | **0.027** | 0.0217 | **0.016** | 0.0499 | **0.011** |
| **Test 3** | 0.0265 | **0.027** | 0.0311 | 0.050 | 0.0195 | 0.064 | 0.0308 | 0.058 |
|  |  | | | |  | | | |
|  | **Weighted UniFrac** | | | | **Unweighted Unifrac** | | | |
|  | Adonis | | Anosim | | Adonis | | Anosim | |
|  | R^2^ | p-value | R statistic | p-value | R^2^ | p-value | R value | p-value |
| **Test 1** | 0.0185 | 0.245 | 0.0060 | 0.268 | 0.0246 | 0.053 | 0.0191 | 0.130 |
| **Test 2** | 0.0189 | 0.244 | 0.0080 | 0.249 | 0.0283 | **0.034** | 0.0266 | 0.068 |
| **Test 3** | 0.0183 | 0.296 | 0.0022 | 0.341 | 0.0261 | **0.046** | 0.0198 | 0.112 |

**SI Table 4. Beta-diversity testing even-numbered Treatment groups.** Beta diversity measures of significant difference between even sample groups (Intervention (n = 34) vs Untreated (n = 34)), tested three different times after the removal of 35 random Intervention samples. All beta diversity metrics with significance are bolded, Bonferroni corrected p-values < 0.05.

Beta diversity metrics were mostly congruent with the results seen in uneven-numbered sample group tests (SI section 1.2). Adonis tests of Bray-Curtis and Unweighted Unifrac measurements of diversity suggest significant differences (SI Table 4; p < 0.05); however, Bray-Curtis adonis (showing significance for variation driven by treatment groups) may be confounded by gender, while Unweighted Unifrac adonis by dentition groups (SI Table 2; see SI section 1.1. for further discussion). Yet Binary Jaccard was no longer significantly different with even-numbered samples (overall), suggesting the measure of presence/absences of species diversity between treatment groups was particularly sensitive to the removal of 35 Intervention samples. This sensitivity is likely related to range of microbial diversity seen in the Intervention group (SI Figure 1.), where loss of 50% of the samples could dramatically impact the presence diversity in the group, as seen by the range of Binary Jaccard p-values (0.016-0.084) across the three tests.

On average, there were no significant differences were detected between the microbial community structure with any distance measure (SI Table 4, anosim, p ≥ 0.05), with the exception of Test 2, which noted significant differences between treatment groups using Bray-Curtis and Binary Jaccard. With the visualisation of Bray-Curtis sample dissimilarities (SI Figure 2), it appears that the significant difference detected between treatment groups in Test 2 (SI Table 4) is an artifact of the removal of those particular 35 samples.

In sum, the removal of 35 children from the Intervention group impacted the consistency of repeated tests, suggesting that the impact of Intervention treatment upon children is variable, and likely unique to each individual child. Therefore, reducing the Intervention group to 34 samples does not capture the true range of variation, and any significant differences of the microbial community structure noted between Intervention and Untreated children should be interpreted with care. As two of the three tests support the uneven-number sample test, we conclude that the Intervention treatment had a minimal impact on the microbial community structure of salivary microbiota. Overall, the inconsistency of beta-diversity tests of even-numbered samples indicates that results should be treated with circumspection, and no definitive conclusions could be drawn from this test without further information.


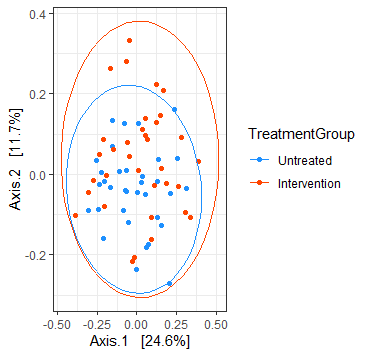

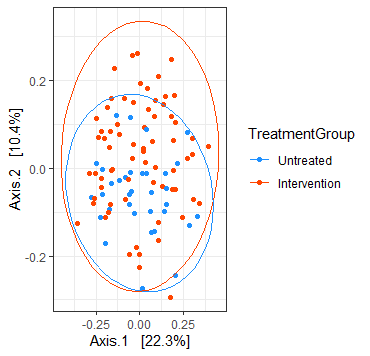


A

B

**SI Figure 2. Principle coordinate analysis (PCoA) plots from Bray -Curtis beta diversity distance matrices at the feature level. (A)** Removal of 35 random samples in Test 2 of Intervention (n = 34) vs Untreated (n = 34) induced a significant difference between samples groups (anosim, p = 0.03, R = 0.044), whereas **(B)** uneven sample groups of Intervention (n = 69) vs Untreated (n = 34) was found not significant (anosim, p = 0.70, R = -0.019). All samples are plotted in three-dimensional space relative to their dissimilarity to one another.

### 1.4.2. Caries-Active Intervention and Caries-Active Untreated groups

As there is a very low number of CF Untreated children (n = 5), we first focused on balancing the sampling groups of only CA children (CA Intervention (n = 38) vs CA Untreated (n = 29)) to confirm the impact of the Intervention treatment on CA microbial community. As before, nine random CA Intervention sample (nine different individuals) were removed and all analysis were repeated to identify the alpha and beta diversity differences between children. This was done three different times, each time removing nine different random samples to create three different datasets (Test 1, Test 2, and Test 3) looking at the alpha and beta diversity differences between an equal number CA children (CA Intervention (n = 29) vs CA Untreated (n = 29)).

All alpha diversity metrics, across the three different tests, were congruent with the findings seen in the unequal sample groups, supporting the greater microbial diversity in children who did not receive Intervention treatment (SI Table 5, p < 0.02).

|  | **CA Intervention (n = 29) vs CA Untreated (n = 29)** | | | | | |
| --- | --- | --- | --- | --- | --- | --- |
|  | **Shannon** | | **Observed species** | | **Chao1** | |
|  | T statistic | p-value | T statistic | p-value | T statistic | p-value |
| **Test 1** | 3.2685 | **0.002** | 2.8206 | **0.007** | 2.6259 | **0.009** |
| **Test 2** | 3.6886 | **0.001** | 3.6446 | **0.003** | 3.4363 | **0.002** |
| **Test 3** | 3.3550 | **0.003** | 2.9679 | **0.003** | 2.6211 | **0.024** |

**SI Table 5. Testing even-numbered sample groups.** Alpha diversity measures of significant differences between even sample groups (CA Intervention (n = 29) vs CA Untreated (n = 29)), tested three different times after the removal of nine random CA Intervention samples. All alpha diversity metrics with significance are bolded, Bonferroni corrected p-values < 0.05.

Beta diversity metrics were also congruent with the results seen in uneven sample group tests, with no significant differences were detected between the microbial community structure with any distance measure (SI Table 6, anosim, p ≥ 0.05). However, differences were detected in the amount of microbial variation that could be attributed to presence/absence measures of beta diversity; unweighted Unifrac no longer supported significant differences (SI Table 6, adonis, p ≥ 0.08). However, binary Jaccard still supports significance differences between groups attributed to the non-phylogenetic presence/absence measures of microbial variation in two of the three tests (SI Table 6). Overall, with limited congruence to the uneven sample groups, these results suggest that the Intervention did not impact the overall microbial community structure of CA children.

|  | **CA Intervention (n = 29) vs CA Untreated (n = 29)** | | | | | | | |
| --- | --- | --- | --- | --- | --- | --- | --- | --- |
|  | **Bray-Curtis** | | | | **Binary Jaccard** | | | |
|  | Adonis | | Anosim | | Adonis | | Anosim | |
|  | R^2^ | p-value | R statistic | p-value | R^2^ | p-value | R value | p-value |
| **Test 1** | 0.0268 | 0.08 | 0.0183 | 0.16 | 0.0232 | **0.04** | 0.0416 | 0.06 |
| **Test 2** | 0.0291 | 0.05 | 0.0196 | 0.15 | 0.0236 | **0.03** | 0.0287 | 0.09 |
| **Test 3** | 0.0247 | 0.11 | 0.0116 | 0.24 | 0.0219 | 0.06 | 0.0280 | 0.10 |
|  |  | | | |  | | | |
|  | **Weighted UniFrac** | | | | **Unweighted Unifrac** | | | |
|  | Adonis | | Anosim | | Adonis | | Anosim | |
|  | R^2^ | p-value | R statistic | p-value | R^2^ | p-value | R value | p-value |
| **Test 1** | 0.0276 | 0.17 | 0.0255 | 0.11 | 0.0263 | 0.08 | -0.0007 | 0.46 |
| **Test 2** | 0.0260 | 0.18 | 0.0098 | 0.25 | 0.0271 | 0.08 | 0.0074 | 0.30 |
| **Test 3** | 0.0206 | 0.30 | 0.0131 | 0.18 | 0.0252 | 0.10 | 0.0132 | 0.23 |

**SI Table 6. Testing even-numbered sample groups.** Beta diversity measures of significant difference between even sample groups (CA Intervention (n = 29) vs CA Untreated (n = 29)), tested three different times after the removal of nine random CA Intervention samples. All beta diversity metrics with significance are bolded, Bonferroni corrected p-values < 0.05.

## *1.5 Severity of caries is related to oral microbial composition*

Given the minimal impact of presence or absence of dental caries on oral microbial diversity, we looked to examine how caries presence and severity influences the oral microbiota—regardless of treatment—by combining all children into three levels of caries: no caries (*i.e.* no carious lesions detected, score of 0–2 (n = 36)), moderate dental caries (ICDAS 3–4; n = 33), and severe caries (ICDAS 5–6; n = 34). No differences in microbial diversity were detected across children with varying levels of caries (alpha diversity; SI Table 7). However, significant microbial community differences were detected for presence/absence measures of diversity (binary Jaccard adonis, p = 0.03, R^2^ = 0.025; anosim, p = 0.02, R = 0.034; unweighted Unifrac adonis, p = 0.03, R^2^ = 0.032, anosim, p = 0.01, R = 0.0367), but not for abundance-based measures of diversity (Bray-Curtis adonis, p = 0.13, R^2^ = 0.026; anosim, p = 0.138, R = 0.014; weighted Unifrac adonis, p = 0.40, R^2^ = 0.0198; anosim, p = 0.55, R = -0.0050). Without accounting for treatment group, which we noted to impact microbial diversity, it is likely the presence/absence measures of microbial community differences is effected by Intervention and Untreated diversity differences; especially in that Intervention samples contribute to the majority (86%) of the no caries sample size.

| **Caries Severity** | **Shannon** | | **Observed species** | | **Chao1** | |
| --- | --- | --- | --- | --- | --- | --- |
|  | T statistic | p-value | T statistic | p-value | T statistic | p-value |
| no caries *vs* moderate caries | -0.9114 | 1 | -1.0128 | 0.98 | -0.8775 | 1 |
| severe caries *vs* moderate caries | -0.3610 | 1 | -0.9413 | 1 | -0.6267 | 1 |
| no caries *vs* severe caries | 0.4978 | 1 | 0.1877 | 1 | 0.3233 | 1 |

**SI Table 7.** Alpha diversity statistics for caries severity groups, not accounting for impact of treatment group. Bonferroni corrected p-values < 0.05; no significance was detected.

**Additional Files – SI Table Legends**

**Additional File 2**

**SI Table 8. Three species detected significantly greater in Untreated group (Kruskal-Wallis test).** Significant differences (Kruskal-Wallis) between treatment groups, not accounting for dental caries. Bolded p-values are FDR corrected and significant p < 0.05. Unique 16S rRNA sequences were assigned species taxonomy using the Human Oral Microbiome Database (HOMD; v. 15.1; Chen et al. 2010), Greengenes database (v. 13.8; DeSantis et al. 2006), and ribosomal database SILVA (132 release; Glöckner et al. 2017), after testing for significance.

**Additional File 3**

**SI Table 9. Nine species significantly different between three levels of caries severity (Kruskal-Wallis test).** Significant differences (Kruskal-Wallis) between caries severity, not accounting for treatment groups. Bolded p-values are FDR corrected and significant p < 0.05. Unique 16S rRNA sequences were assigned species taxonomy using the Human Oral Microbiome Database (HOMD; v. 15.1; Chen et al. 2010), Greengenes database (v. 13.8; DeSantis et al. 2006), and ribosomal database SILVA (132 release; Glöckner et al. 2017), after testing for significance

**Additional File 4**

**SI Table 10. Nine species detected significantly different (Kruskal-Wallis test) between treatment groups when accounting for dental caries severity.** Significant differences (Kruskal-Wallis) between treatment groups and caries severity. Bolded p-values are FDR corrected and significant p < 0.05. Unique 16S rRNA sequences were assigned species taxonomy using the Human Oral Microbiome Database (HOMD; v. 15.1; Chen et al. 2010), Greengenes database (v. 13.8; DeSantis et al. 2006), and ribosomal database SILVA (132 release; Glöckner et al. 2017), after testing for significance

**Additional File 5**

**SI Table 11. Sample and sequencing information.**

# References

**Table 6. Kruskal-Wallis Group Significance of caries severity between treatment groups**. All in-text p-values reporting FDR-corrected p-value. QIIME2 feature ID assigned to three difference 16S rRNA databases: Greengenes, SILVA, and HOMD.

1. Crielaard W, Zaura E, Schuller AA, Huse SM, Montijn RC, Keijser BJ. Exploring the oral microbiota of children at various developmental stages of their dentition in the relation to their oral health. BMC Medical Genomics. 2011;4:22.

2. Mason MR, Chambers S, Dabdoub SM, Thikkurissy S, Kumar PS. Characterizing oral microbial communities across dentition states and colonization niches. Microbiome. 2018;6:67.

3. Langdon A, Crook N, Dantas G. The effects of antibiotics on the microbiome throughout development and alternative approaches for therapeutic modulation. Genome Medicine. 2016;8:39.

4. Glöckner FO, Yilmaz P, Quast C, Gerken J, Beccati A, Ciuprina A, et al. 25 years of serving the community with ribosomal RNA gene reference databases and tools. Journal of Biotechnology. 2017;261:169–76.

5. Chen T, Yu W-H, Izard J, Baranova OV, Lakshmanan A, Dewhirst FE. The Human Oral Microbiome Database: a web accessible resource for investigating oral microbe taxonomic and genomic information. Database. 2010;2010.

6. DeSantis TZ, Hugenholtz P, Larsen N, Rojas M, Brodie EL, Keller K, et al. Greengenes, a Chimera-Checked 16S rRNA Gene Database and Workbench Compatible with ARB. Appl Environ Microbiol. 2006;72:5069–72.
